# High frequency of the *exoU*+/*exoS*+ genotype associated with multidrug-resistant “high-risk clones” of *Pseudomonas aeruginosa* clinical isolates from Peruvian hospitals

**DOI:** 10.1038/s41598-019-47303-4

**Published:** 2019-07-26

**Authors:** Gertrudis Horna, Catherine Amaro, Aida Palacios, Humberto Guerra, Joaquim Ruiz

**Affiliations:** 1 Barcelona Institute for Global Health, ISGlobal, Hospital Clinic - Universitat de Barcelona, Barcelona, Spain; 2 Universidad Catolica los Angeles de Chimbote, Instituto de Investigacion, Chimbote, Peru; 3 Universidad Peruana Cayetano Heredia, Instituto de Medicina Tropical Alexander von Humboldt, Lima, Peru; 4 Hospital Nacional Cayetano Heredia, Lima, Peru; 5 Universidad Continental, Lima, Peru

**Keywords:** Antimicrobial resistance, Molecular biology

## Abstract

The type III secretion system of *Pseudomonas aeruginosa* is an important virulence factor contributing to the cytotoxicity and the invasion process of this microorganism. The current study aimed to determine the presence of the *exoU*+/*exoS*+ genotype in *P*. *aeruginosa* clinical isolates. The presence of *exoS*, *exoT*, *exoU* and *exoY* was determined in 189 *P*. *aeruginosa* by PCR, and the presence/absence of *exoU* was analysed according to source infection, clonal relationships, biofilm formation, motility and antimicrobial susceptibility. The *gyrA*, *parC*, *oprD*, efflux pump regulators and β-lactamases genes were also analysed by PCR/sequencing. The *exoS*, *exoT* and *exoY* genes were found in 100% of the isolates. Meanwhile, *exoU* was present in 43/189 (22.8%) of the isolates, being significantly associated with multidrug resistance, extensively drug resistance as well as with higher level quinolone resistance. However, the presence of β-lactamases, mutations in *gyrA* and *parC*, and relevant modifications in efflux pumps and OprD were not significantly associated with *exoU*+ isolates. MLST analysis of a subset of 25 isolates showed 8 different STs displaying the *exoU*+/*exoS*+ genotype. The MDR basis of the *exoU*+ isolates remain to be elucidated. Furthermore, the clinical implications and spread of *exoU*+/*exoS*+ *P*. *aeruginosa* isolates need to be established.

## Introduction

*Pseudomonas aeruginosa* is a Gram-negative pathogen causing opportunistic infections in susceptible hosts. It is a leading cause of acute pneumonia in hospitalised patients and is responsible for chronic lung infections in patients with cystic fibrosis^1^. One of the reasons for the poor clinical outcomes of *P*. *aeruginosa* infections is thought to be virulence factors, especially the Type III secretion system (T3SS) which is considered an important contributor to cytotoxicity and the invasion process^2–4^. This system allows these bacteria to directly inject effector proteins into eukaryotic cells. At present, four effector proteins have been identified: ExoU, a phospholipase; ExoY, an adenylate cyclase; and ExoS and ExoT, which are bifunctional proteins. ExoT and ExoY are encoded by almost all strains, therefore might be considered an inevitable component of *P*. *aeruginosa* virulence^5^. ExoS and ExoU contribute greatly to pathogenesis. Thus, ExoU is responsible for a highly cytotoxic phenotype which leads to host cell death and is considered to be a relevant factor involved in the severity of infections and as an independent factor of early mortality during blood infections^6–8^. Furthermore, it has been shown that the *exoU* gene is a key factor in the early stages of *P*. *aeruginosa* pneumonia^9^. Meanwhile, the distribution of the genes encoding these effectors is not uniform and some, particularly *exoS* and *exoU*, are almost always mutually exclusive^5,7,10–12^. probably because these genes provide enhanced fitness in distinct ecological niches^13^. However, some reports have shown the concomitant presence of both genes in a significant number of clinical isolates^14–16^.

In recent years, the incidence of multidrug resistance (MDR) especially to fluoroquinolones (FQs) and carbapenems, has increased, becoming a major issue for nosocomial infection by *P*. *aeruginosa*. In this microorganism, the mechanisms of resistance to FQs are mainly chromosomal such as the presence of target-site gene mutations (TSMs) or increased production of resistance–nodulation–cell division (RND) type efflux pumps^4,17^. However, quinolone resistance transferable determinants such as the presence of *qnr* or *crpP* genes have been reported^18,19^. On the other hand, the most frequent mechanisms of resistance to carbapenems include the inactivation of OprD, the increased production of multidrug efflux pumps, and hydrolysis by carbapenemases^4,17^.

Several studies have reported the relationship between the presence of the *exoU* gene and MDR in clinical isolates of *P*. *aeruginosa*^3,4,15,20^. However, the concomitant presence of the *exoU*+ and *exoS*+ genes has scarcely been reported due to the frequent mutual exclusion of the two genes. Therefore, this study aimed to determine the presence of the *exoU*+/*exoS*+ genotype and its association with phenotypic characteristics, resistance genes related to MDR and efflux pump regulators in clinical isolates of *P*. *aeruginosa*.

## Results

### Bacterial isolates and distribution of genes encoding T3SS

The *exoS*, *exoT* and *exoY* genes were found in 100% of the isolates studied. Meanwhile, the *exoU* gene was present in 43/189 (22.8%) of the isolates, Therefore, all isolates presenting the *exoU*+ gene were *exoU*+/*exoS*+.

No unspecific annealing of the primers was detected during the *in silico* analysis, thereby ruling out the possibility of false priming. On the other hand, the high prevalence of *exoU*+ genotype isolates was not related to the spread of a unique BOX-pattern. Thus, *exoU*+ isolates were classified within 25 different BOX groups. Sixteen BOX-patterns contained both *exoU*+ and *exoU−* genes (Supplementary Figure). The results were fully validated by the sequencing of *exoU* and *exoS* amplicons in 25 isolates (one for each BOX group).

### exo*U*+/*exoS*+ genotype and hospital wards

The *exoU* gene was detected in 23/77 (53.4%) of the isolates from Hospital Arzobispo Loayza (HAL) and in 20/112 isolates (46.5%) of isolates from Hospital Nacional Cayetano Heredia (HNCH), bordering but without reaching significant differences (*p* = 0.0529).

Fifty-three *P*. *aeruginosa* had no data about hospital ward origin. The analysis of the remaining 136 isolates showed that *exoU* was more frequent among *P*. *aeruginosa* from patients attending Intensive Care Units (ICUs) [9/18 (50.0%) *p* = 0.0197] and the burn ward (6/8, 75.0%, *p* = 0.0019). Nonetheless, the presence of *exoU* was not specifically associated with any source of infection (Tables 1 and 2).

**Table 1:** Distribution of the *exoU*+/*exoS*+ genotype among the isolates analyzed.

Characteristics	*exoU*+/*exoS*+	*exoU−/exoS*+
Number of isolates = 189	43	146
HNCH isolates = 112	20 (46.5%)	92 (63.0%)
HAL isolates = 77	23 (53.4%)	54 (36.9%)
Bronchial secretions 72/189	15 (34.8%)	57 (39.0%)
Wounds/abscesses = 33/189	10 (23.2%)	23 (15.7%)
Urine = 55/189	13 (30.2%)	42 (28.7%)
Other = 29/189	5 (11.6%)	24 (16.4%)
SBP = 78	14 (32.5%)	64 (43.8%)
Presence of twitching = 163	38 (88.3%)	125 (85.6%)
Presence of swarming = 109	21(48.8%)	88 (60.2%)
Presence of swimming = 157	36 (83.7%)	121 (82.8%)

HNCH: Hospital Nacional Cayetano Heredia, HAL: Hospital Arzobispo Loayza, SBP: Strong biofilm producer. In no case were observed significant differences.

**Table 2:** Hospital ward origin of *Pseudomonas aeruginosa isolates*.

Ward	n = 189	*exoU*+/exoS+	*exoU−/exoS+*
ICU	18	9 (50.0%)^b^	9 (50.0%)
Burns^a^	8	6 (75.0%)^c^	2 (25.0)
External	28	9 (32.1%)	19 (67.9%)
Other	82	13 (15.9%)	69 (84.1%)
ND	53	6 (12.8%)	47 (87.2)

ICU: Intensive Care Units; Externa: Samples belonging to hospital outpatients; ND: Isolates from which no specific hospital ward data was recorded, thus these isolates have not been included in the statiscal analysis; Other: All remaining hospitalisation wards.

^a^All burn patients were from Hospital Arzobispo Loayza.

^b^Significant number of *exoU*+ isolates (*p* = 0.0197).

^c^Significant number of *exoU*+ isolates (*p* = 0.0017).

### exo*U*+/*exoS*+ genotype and biofilm production and bacterial motility

On analysing the association of the presence of *exoU*+/*exoS*+ genotype with other bacterial characteristics, it was observed that 42/43 (97.7%) *exoU*+/*exoS*+ and 142/146 (97.3%) *exoU−*/*exoS*+ isolates were able to form biofilm, although no significant association with strong biofilm production was detected. Thus 14/43 (32.5%) *exoU*+ isolates presented strong biofilm production (SBP), while 64/146 (43.8%) *exoU−* isolates presented the SBP phenotype (Table 1). With respect to bacterial motility, there was no significant association between *exoU* and the motility phenotype; however, 88.3%, 83.7% and 48.8% *exoU*+ isolates presented twitching, swimming and swarming, respectively (Table 1).

### exo*U*+/*exoS*+ genotype and antimicrobial resistance

**Figure 1:**
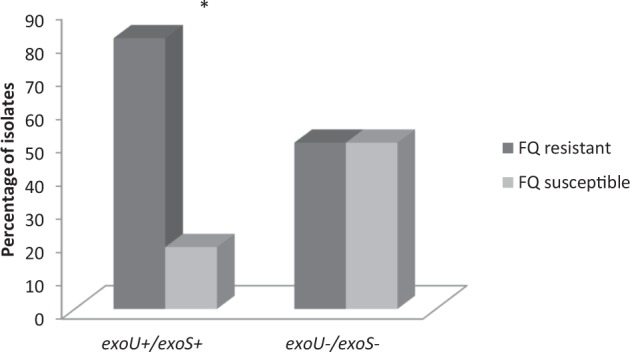
Levels of non-susceptibility to fluoroquinolones in *exoU*+/*exoU−* isolates. FQ: Fluoroquinolones; *p = 0.000256.

The *exoU*+ genotype was more likely to be found in FQ and aminoglycoside non-susceptible than susceptible isolates. Thus, 35/43 (81.4%) FQ non-susceptible isolates showed the presence of the *exoU* gene (*p* = 0*.*000256) (Fig. 1). Similarly, 30/43 (69.8%) of the *exoU*+ isolates were non-susceptible to aminoglycosides (*p* = 0.001246). On the other hand, although not significant, the *exoU*+ isolates were more resistant to cephalosporins and carbapenems. Thus, 24/43 (55.9%) and 27/43 (62.8%) of the isolates displaying the *exoU*+ genotype were non-susceptible to these antimicrobial classes, respectively (Table 3).

**Table 3:** Percentage of antimicrobial resistance of *Pseudomonas aeruginosa* isolates.

Antimicrobial Agents	Total (n = 189)		
	*exoU*+/*exoS*+ (n = 43)	*exoU−/exoS*+ (n = 146)	*P value*
Cephalosporins	55.8	39.7	0.061364
CAZ	44.2	37.0	0.394074
FEP	55.8	39.0	0.050771
Monobactam			
ATM	69.8	38.4	**0.000277**
β-lactam + inhibitors			
PTZ	41.9	32.9	0.277429
Carbapenems	62.8	48.6	0,102388
IMI	60.5	47.9	0.148927
MER	62.8	41.8	**0.015202**
Aminoglycosides	69.8	41.8	**0.001246**
GM	65.1	41.1	**0.005514**
TO	34.9	41.1	0.464281
AK	60.5	36.3	**0.004749**
Fluoroquinolones	81.4	50.0	**0.000256**
CIP	76.7	43.8	**0.000148**
LVX	81.4	45.2	**0.000029**
OFX	81.4	49.3	**0.000191**
Polymyxins	0	0	—
COL	0	0	—
PS	16.3	34.2	**0.024049**
MR	7.0	22.6	**0.021821**
MDR	20.9	8.2	**0.019749**
XDR	55.8	34.9	**0.013893**
MDR + XDR	76.7	43.1	**0.000108**

CAZ: Ceftazidime, FEP: Cefepime, ATM: Aztreonam, PTZ: Piperacillin/Tazobactam, IMI: Imipenem, MER: Meropenem, GM: Gentamicin, TO: Tobramicin, AK: Amikacin, CIP: Ciprofloxacin, LVX: Levofloxacin, OFX: Ofloxacin. PS: Pan-susceptible. MR: Moderately drug-resistant; MDR: Multi drug-resistant. XDR: Extensively drug-resistant. The significant differences are highlighted in bold.

On analysing the resistance levels to specific antibacterial agents, the *exoU*+ isolates were related to higher levels of resistance to all the FQs (levofloxacin - LVX; 81.4%, *p* = 0.000029; ofloxacin - OFX: 81.4%, *p* = 0.000191; ciprofloxacin - CIP: 76.7%. *p* = 0.000148) and monobactams (aztreonam: 69.8%, *p* = 0.000277) tested, as well as several aminoglycosides (gentamicin: 65.1%, *p* = 0.005514 and amikacin: 60.5%, *p* = 0.004749) and carbapenems (meropenem: 62.8%, *p* = 0.015202). Overall, 33 out of 43 *exoU*+ isolates were classified as MDR or XDR [extensively drug-resistant] (*p* = 0.000108). Thus, the presence of *exoU* was associated with both MDR (20.9%, *p* = 0.019749) and XDR (55.8%, *p* = 0.013893) isolates. On the other hand, the absence of *exoU* was associated with pan-susceptible (PS) and moderately drug-resistant (MR) isolates (34.2%, *p* = 0.024049 and 22.6%,* p* = 0.021821 respectively) [Table 3]. All the isolates were susceptible to colistin.

### exo*U*+/*exoS*+ genotype and non-susceptibility to fluoroquinolones

**Figure 2:**
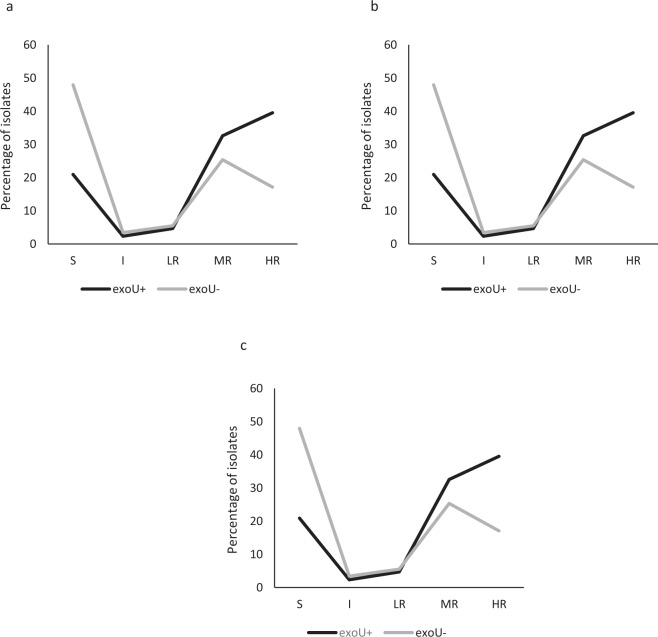
Association between fluoroquinolone MIC levels and *exoU*+/*exoU−* isolates. Only significant differences are reported. (**a**) Differences in the levofloxacin MIC levels between *exoU*+ and *exoU−* isolates. S: Susceptible (MIC ≤ 2 mg/L; *p* = 0.001596); I: Intermediate (MIC of 4 mg/L); LR: Low Resistance Levels (MIC of 8–16 mg/L); MR: Moderate Resistance Levels (MIC of 32–64 mg/L); HR: High Resistance Levels (MIC > 128 mg/L; *p* = 0.002818). (**b**) Differences in the ofloxacin MIC levels between *exoU*+ and *exoU−* isolates. S: Susceptible (MIC ≤ 2 µg/ml; *p* = 0.003488); I: Intermediate (MIC of 4 µg/ml); LR: Low Resistance Levels (MIC of 8–16 mg/L); MR: Moderate Resistance Levels (MIC of 32–64 mg/L); HR: High Resistance Levels (MIC > 128 mg/L; *p* = 0.004902). (**c**) Differences in the ciprofloxacin MIC levels between *exoU*+ and *exoU−* isolates. S: Susceptible (MIC ≤ 1 mg/L; p = 0.000859); I: Intermediate (MIC of 2 mg/L); LR: Low Resistance Levels (MIC of 4–8 mg/L); MR: Moderate Resistance Levels (MIC of 16–32 mg/L); HR: High Resistance Levels (MIC > 64 mg/L; *p* = 0.000191).

To further evaluate the correlation between the level of susceptibility to FQ and the T3SS genotype, the presence of the *exoU*+ genotype regarding distribution of the MIC of LVX, OFX and CIP was determined (Fig. 2). The results showed that the presence of *exoU*+ was significantly more frequent among isolates with a high level of resistance (MIC > 128 mg/L) to LVX/OFX (*p* = 0.002818/*p* = 0.004902 respectively) and to CIP (MIC > 64 mg/L; *p* = 0.000191), while no differences were found between *exoU*+ and *exoU−* genotypes regarding low or moderate resistance levels to any of the FQ tested. On the other hand, *exoU−* isolates were associated with FQ susceptibility [LVX/OFX (MIC < 2 mg/L; *p*-values: 0.001596/0.003488) and CIP (MIC < 1 mg/L; *p* = 0.000859)] (Fig. 2).

### exo*U*+/*exoS*+ genotype and mutations in target genes of QRDR

The *gyrA* and *parC* QRDR regions were amplified and sequenced in a subset of 50 isolates; 13/43 (30.2%) *exoU*+ and 37/146 (25.3%) *exoU−* (Table 4). Of these, 2 *exoU*+ and 14 *exoU−* isolates had no TSM. However, 5 of these 14 *exoU−* isolates (1094, 1104, 1117, 1120 and 1121) displayed resistance to at least one of the FQs tested.

**Table 4:** Distribution of *exoU*+/*exoS*+ genotype according to the *gyrA/parC* QRDR, *oprD* gene and efflux pumps regulators.

	*exoU*+ (n = 13)%	*exoU−* (n = 37)%	*P value*
TSM single	2 (15.3)	5 (13.5)	0.867
TSM multiple	9 (69.2)	18 (48.6)	0.200
MexAB - RM	5 (38.5)	21 (56.7)	0.256
MexAB - IM	8 (61.5)	16 (43.2)	
MexEF- RM	4 (30.7)	19 (51.3)	0.200
MexEF- IM	9 (69.2)	18 (48.6)	
MexXY - RM	1 (7.70)	2 (5.40)	0.765
MexXY - IM	12 (92.3)	35 (94.6)	
*oprD* - RM	7 (53.8)	17 (45.9)	0.623
*oprD* - IM	6 (46.1)	20 (54.0)	

TSM: Target Site Mutation RM: Relevant Modification; IM: Irrelevant Modification.

No alteration was found on *nfxB* (MexCD regulator).

Similar proportions of single TSM were observed between *exoU*+ and *exoU−* isolates. Thus 2/13 (15.3%) *exoU*+ and 5/37 (13.5%) *exoU−* isolates possessed a single TSM. Meanwhile, 9/13 (69.2%) of *exoU*+ and 18/37 (48.6%) of *exoU−* isolates possess multiple TSMs (Tables 4 and 5).

**Table 5:** Modifications in target genes of the QRDR, efflux pump regulators, the *oprD* gene and antimicrobial susceptibility to fluoroquinolones and carbapenems in *exoU*+ and *exoU−* isolates.

*exoU* Genotype	Isolates	*gyrA*	*parC*	*nalD*	*nalC*	*mexR*	*nfxB*	*mexZ*	*mexS*	*mexT*	FQ MIC (mg/L)			*oprD*	IMI	MER
											LVX	OFX	CIP			
*exoU+*	1069	—	—	—	G71E	—	—	—	NA-c	—	0.5	2	0.5	2	S	S
*exoU+*	1070	T83I	S87L	∆_nt397-398_	G71E,S209R	V126E	—	L105R	—	—	64	128	32	W65* + 2	R	R
*exoU+*	1071	T83I	S87L	—	G71E,S209R	V126E	—	L105R	—	—	64	128	32	W65* + 2	R	R
*exoU+*	1072	T83I	S87L	NA	G71E,S209R	V126E,L131P	—	L105R	NA-b	—	64	128	64	NA	R	R
*exoU+*	1073	E153K	—	R44P	G71E,S209R,A145V	V126E	—	L105R,R167S	—	—	32	64	8	3	S	S
*exoU+*	1074	—	—	—	G71E,S209R	V126E	—	NA	NA-c	—	1	2	0.5	3	S	S
*exoU+*	1075	T83I	S87L	—	G71E,S209R,D79E	V126E	—	G56S	V73A	—	16	64	16	3	R	R
*exoU+*	1076	—	S87L	—	G71E,S209R	—	—	—	—	—	128	>256	128	T103S,K115T,F170L, E185Q,P186G,V189T,G307D	R	R
*exoU+*	1077	T83I	S87L	—	G71E,S209R	V126E	—	—	—	—	64	32	64	ins_nt 1201-1205_ (GTCCA) + 3	R	R
*exoU+*	1078	T83I	S87L	∆_nt 263-279_	—	V126E	—	—	—	—	128	256	64	ins_nt 1201-1205_ (GTCCA) + 3	R	R
*exoU+*	1079	T83I/D87N	S87L	∆_nt 263-279_	G71E,S209R	V126E	—	—	—	—	256	256	256	3	S	S
*exoU+*	1080	T83I	S87L	∆_nt 263-279_	G71E,S209R	V126E	—	—	NA-b	-	64	128	64	ins_nt 1201-1205_ (GTCCA) + 3	R	R
*exoU+*	1081	T83I	S87L	L194R	G71E,S209R	V126E	—	—	—	—	64	256	64	3	S	S
*exoU−*	1082	—	—	—	G71E,S209R	—	—	—	—	NA	0.5	1	0.5	4	S	S
*exoU−*	1084	T83I	S87L	NA	G71E,A145V,S209R	—	—	—	NA-c	P185R	128	256	64	ins_nt941–942_(GC)	R	R
*exoU−*	1085	T83I	S87L	∆_nt451-461_	G71E,S209R	—	—	Y18C	NA-c	—	128	256	128	—	S	S
*exoU−*	1086	T83I	S87L	∆_nt 397-398_	G71E,S209R	V126E	—	∆_AA F103-V112_	—	—	64	128	64	ins_nt678_ (G) + C	R	R
*exoU−*	1087	—	—	—	NA	V126E	—	L105R	NA-c	—	1	2	0.25	3	R	S
*exoU−*	1088	—	—	—	NA	V126E	—	—	NA-c	NA	0.5	1	0.5	2	S	S
*exoU−*	1089	T83I	S87L	∆_nt 397-398_	G71E,S209R	V126E	—	L105R	—	—	128	128	32	2	R	R
*exoU−*	1092	D87N	S87L	1	G71E,S209R	V126E	—	S66L	—	—	4	4	2	NA	S	S
*exoU−*	1093	T83I	—	∆_nt 397-398_	G71E,S209R	V126E	—	L105R	—	—	64	256	32	NA	R	R
*exoU−*	1094	—	—	—	∆_nt234-243_ + G71E,Q81P	—	—	—	NA-b	—	4	16	0.5	2	S	S
*exoU−*	1095	T83I	S87L	—	G71E,S209R	NA	—	H18Y	NA-b	—	64	64	32	Y49* + 4	R	R
*exoU−*	1096	T83I	S87L	∆_nt 391_	G71E,S209R	V126E	—	V72L	G224S	—	128	128	64	V127L	S	S
*exoU-*	1097	—	—	∆_nt 391_	G71E,S209R	—	—	—	NA-b	—	4	2	0.25	3	S	S
*exoU−*	1098	—	—	T188A	G71E,S209R,P210L	—	—	Q21*	—	—	1	1	1	ins_nt 605-609_ (CAACA) + 4	R	S
*exoU−*	1100	T83I	S87L	—	G71E,S209R	NA	—	H18Y	—	—	64	128	32	Y49* + 4	R	R
*exoU−*	1102	T83I	S87L	—	G71E,S209R	V126E	—	H18Y	—	—	16	64	8	Y49* + 4	R	R
*exoU−*	1103	—	—	—	G71E,A186T	—	—	E124K	—	—	2	2	0.25	NA	S	S
*exoU−*	1104	—	—	—	G71E,S209R	L131P	—	—	NA-c	—	8	32	32	4	R	R
*exoU−*	1105	T83I	S87L	∆_nt 397-398_	G71E,S209R	V126E	—	L105R	—	—	64	64	32	W65* + 2	R	R
*exoU−*	1106	—	S87L	—	—	NA	—	∆_nt408-409_	NA-c	—	4	2	1	—	S	S
*exoU−*	1107	—	—	—	G71E,S209R,P210L	NA	—	L95M	NA-c	D290E	2	2	0.5	4	S	S
*exoU−*	1108	—	—	T188A	G71E,S209R	—	—	—	—	—	0.5	2	0.5	3	S	S
*exoU−*	1109	—	—	—	G71E,S209R,D79E	—	—	—	—	NA	0.5	1	0.5	4	S	S
*exoU−*	1110	T83I	S87L	—	G71E,S209R	—	—	—	—	P185R	128	256	128	ins_nt941–942_(GC)	R	S
*exoU−*	1111	T83I	S87L	—	—	NA	—	H18Y	NA-b	—	128	> 256	128	Y49* + 4	R	R
*exoU−*	1112	T83I	S87L	—	G71E,S209R	NA	—	H18Y	NA-b	—	64	128	64	Y49* + 4	R	R
*exoU−*	1113	T83I	S87L	∆_nt 397-398_	G71E,S209R	V126E	—	L105R	—	—	128	128	32	W65* + 2	R	R
*exoU−*	1114	—	S87L	—	G71E,S209R	—	—	—	—	—	16	32	16	4	S	S
*exoU−*	1115	T83I	S87L	—	G71E,S209R	—	—	—	—	—	32	64	32	4	S	S
*exoU−*	1116	T83I	S87L	—	G71E,S209R	—	—	—	—	—	32	64	32	4	S	S
*exoU−*	1117	—	—	—	—	—	—	—	NA-c	—	16	4	0.25	4	S	S
*exoU−*	1118	T83I/D87N	S87L	∆_nt 263-279_	G71E,S209R	V126E	—	—	—	—	128	>256	>256	3	S	S
*exoU−*	1119	T83I	—	—	S209R	V126E	—	—	NA-b	—	8	16	4	4	S	S
*exoU−*	1120	—	—	—	G71E,S209R	V126E	—	—	NA-d	—	8	4	2	4	S	S
*exoU−*	1121	—	—	—	G71E,S209R	NA	—	L141Q	NA-c	—	8	64	1	ins_nt1087_(A) + ∆_nt 1294_(T) + T103S,K115T,F170L	R	R
*exoU−*	1122	D87N	—	—	G71E,S209R	—	—	—	NA-c	—	64	64	64	Y49* + 4	R	R
*exoU−*	1123	T83I	S87L	—	G71E,S209R	NA	—	H18Y	—	—	64	128	32	Y49* + 4	R	R

The symbol “−” represent wild type isolates; NA: No amplification; the symbol ∆_nt_ represents nucleotide deletion being noted the first and last nucleotides deleted; the symbol ∆_AA_ represents amino acid deletions being noted the first and last amino acid deleted; ins_nt_: nucleotide insertion; The symbol “*” represents codon STOP. FQ-MIC: MIC to fluoroquinolones (LVX: Levofloxacin; OFX: Ofloxacin; CIP: Ciprofloxacin); IMI/MER are isolates showing susceptibility (S); resistance or intermediate susceptibility (R) to imipenem or meropenem performed by the disk diffusion assay.

Alterations at *nalC*, *nalD*, *mexR*, *mexS* and *mexT* as well as the patterns of OprD were previously described^17^. The patterns of OprD are named accordingly to Horna *et al*.^17^.

1. Amino acid substitutions: Q134H, Q142H, A145P, D147H, E148K, C149R, H154P, R160K, D176E, D185Y, G206S, S209I.

2. *oprD*-Pattern C^17^: V127L, E185Q, P186G, V189T, E202Q, I210A, E230K, S240T, N262T, T276A, A281G, K296Q, Q301E, R310E, (±G312R), A315G, L347M, S403A, Q424E + 372V-DSSSSYAGL-383.

3. *oprD*-Pattern B^17^: T103S, K115T, F170L, E185Q, P186G, V189T, R310E, A315G, (±G425A).

4. *oprD*-Pattern A^17^: E202Q, I210A, E230K, S240T, N262T, A267S, A281G, K296Q, Q301E, R310G, (±V352I), V359L, (±Q424R) + 372V-DSSSSYAGL-383.

NA-b: No amplification of *mexS* gene but amplification of N- and C-terminal regions.

NA-c: No amplification of *mexS* gene but amplification of N-terminal region.

NA-d: No amplification of m*exS* gene and no amplification of N- and C- terminal regions.

Overall, 34 isolates with mutations in *gyrA* and/or *parC* showed resistance to at least one FQ. The most frequent amino acid substitutions were T83I and S87L at GyrA and ParC respectively, which were concomitantly found in 24 isolates, and only GyrA T83I was found in 2 isolates and ParC S87L in another 3 isolates. In addition, one isolate showed the amino acid codon substitution D87N in the *gyrA* gene, and another presented the double substitution D87N in GyrA and S87L in ParC. Meanwhile two isolates concomitantly presented 2 amino acid codon substitutions in *gyrA* (T83I/D87N) and one in *parC* (S87L) showing high MICs to all FQs. Finally, one isolate having the GyrA substitution E153K was detected, showing moderate resistance levels to FQ.

### exo*U*+/*exoS*+ genotype and gene regulators of efflux pumps

The analysis of the MexAB-OprM regulators genes (*mexR*, *nalC* and *nalD*) showed the presence of 5/13 (38.5%) relevant and 8/13 (61.5%) irrelevant modifications in the *exoU*+ isolates. Meanwhile these regulators genes showed relevant and irrelevant modifications in the 21/37 (56.7%) and 16/37 (43.2%) of the *exoU−* isolates respectively.

The analysis of the MexEF-OprN regulators shown a similar scenario. Thus, 4/13 (30.7%) and 9/13 (69.2%) of the *exoU*+ displayed relevant and irrelevant modifications in *mexS* and *mexT* respectively. In the same way 19/37 (51.3%) of relevant and 18/37 (48.6%) of irrelevant modifications were detected among *exoU−* isolates. Interestingly, the five *exoU−* isolates (1094, 1104, 1117, 1120, 1121) showing resistance to at least one of the FQs tested in the absence of TSM possess relevant modifications in the *mexS* gene, and also in *nalC* (isolate 1094) and *mexR* (isolate 1121).

Forty-seven out of 50 isolates (94%) showed irrelevant modifications in *mexZ* (a regulator of MexXY-OprM). Regarding MexCD-OprJ, no isolate showed alterations in the *nfxB* gene (Tables 4 and 5).

### exo*U*+/*exoS*+ genotype and β-lactamases

Overall, 67 isolates suspected of carrying metallo-β-lactamases and/or serine-carbapenemases were phenotypically detected: 22 metallo-β-lactamases, 39 serine-carbapenemases and 6 with both metallo-β-lactamases and serine-carbapenemases. It was of note that 4 of these isolates were susceptible to both imipenem and meropenem. PCR results showed that the *bla*_GIM_, *bla*_SIM_, *bla*_SPM_, *bla*_VIM_ genes and *bla*_IMI_ and *bla*_KPC_ corresponding to metallo*-β*-lactamases and serine-carbapenemases, respectively were not found in any isolate. All isolates suspected of carrying metallo-β-lactamases presented *bla*_IMP_ while those which were positive for serine-carbapenemases possessed *bla*_GES_. All the *exoU*+ isolates showed a higher proportion of *bla*_GES_ 32.5% (14/43) than *bla*_IMP_ 9.30% (4/43), however no differences were observed (*p* = 0.246). In contrast, the *exoU−* isolates showed similar proportions of both genes [*bla*_GES_ 21.2% (31/146) vs. *bla*_IMP_ 16.4% (24/146); *p* = 0.125].

### exo*U*+/*exoS*+ genotype and *oprD* gene

Neither relevant nor irrelevant modifications in the *oprD* gene were associated with the presence of *exoU*. Thus, in the *oprD* gene of *exoU*+ isolates were detected 7/13 (53.8%) and 6/13 (46.1%) relevant and irrelevant modifications respectively. Meanwhile, 17/37 (45.9%) and 20/37 (54.0%) *exoU−* isolates showed relevant and irrelevant modifications in *oprD* gene (Tables 4 and 5).

### exo*U*+/*exoS*+ genotype and multi-locus sequence typing (MLST)

MLST analysis showed the presence of 16 different sequence types (STs) among the analysed subset of 25 *P*. *aeruginosa* isolates analysed, with 13 *exoU*+ isolates distributed in 8 different ST patterns. Of these, ST235 and ST357 were the most frequently found. ST235 was detected in 6 isolates from HNCH, with 5 isolates being *exoU*+ (2 XDR, 2 MDR and 1 MR) and 1 *exoU−* (MR). ST357 was detected in 5 isolates from HAL, all being XDR and only susceptible to colistin and with two presenting the *exoU* gene. Furthermore, five new STs were found in the present study (ST3300, ST3301, ST3302, ST3303, and ST3305) two (ST3300 and ST3303) being *exoU*+ [Table 6].

**Table 6:** Distribution of Multi-Locus Sequence Typing and *exoU*+*/exoS*+ genotype.

Isolates	Hospital	*acsA*	*aroE*	*guaA*	*mutL*	*nuoD*	*ppsA*	*trpE*	ST	*exoU* genotype	*β-lactamases*	Antimicrobial profile	Source of infections
1069	HNCH	6	10	5	3	4	3	112	2726	*exoU*+	−	MR	5
1070	HNCH	2	4	5	3	1	6	11	357	*exoU*+	−	XDR*	2
1071	HNCH	2	4	5	3	1	6	11	357	*exoU*+	−	XDR*	3
1072	HNCH	16	4	5	3	1	6	10	**3303**	*exoU*+	*ges* like	XDR	1
1073	HNCH	50	13	105	28	16	7	47	759	*exoU*+	−	MDR	2
1074	HAL	28	5	4	5	4	38	167	**3300**	*exoU*+	−	PS	1
1075	HAL	13	4	5	5	12	7	15	308	*exoU*+	*ges* like	XDR*	1
1076	HAL	22	20	11	3	3	3	7	348	*exoU*+	*ges* like/*imp* like	XDR	2
1077	HAL	38	11	3	13	1	2	4	235	*exoU*+	*ges* like	XDR	3
1078	HAL	38	11	3	13	1	2	4	235	*exoU*+	*ges* like	XDR	3
1079	HAL	38	11	3	13	1	2	4	235	*exoU*+	*ges* like	MDR	2
1080	HAL	38	11	3	13	1	2	4	235	*exoU*+	*imp* like	XDR	1
1081	HAL	38	11	3	13	1	2	4	235	*exoU*+	−	MR	5
1089	HNCH	2	4	5	3	1	6	11	357	*exoU*−	*ges* like*/imp* like	XDR*	5
1093	HNCH	2	4	5	3	1	6	11	357	*exoU*−	*ges* like	XDR*	5
1094	HNCH	28	20	94	5	1	6	10	2118	*exoU*−	−	MR	1
1100	HNCH	36	27	28	3	4	13	7	179	*exoU*−	*ges like/imp* like	MDR	3
1104	HNCH	28	5	5	11	27	15	44	**3302**	*exoU*−	*ges* like	XDR	3
1105	HNCH	2	4	5	3	1	6	11	357	*exoU*−	*ges* like	XDR*	3
1115	HNCH	16	5	11	146	3	4	14	1751	*exoU*−	−	MR	3
1107	HAL	7	5	7	3	4	1	7	**3305**	*exoU*−	*−*	PS	4
1109	HAL	**244**	10	5	5	11	4	7	**3301**	*exoU*−	−	PS	1
1084	HAL	28	5	30	3	4	20	1	699	*exoU*−	*imp* like	MDR	2
1118	HAL	38	11	5	13	1	2	4	235	*exoU*−	−	MR	3
1119	HAL	6	5	6	5	4	4	7	641	*exoU*−	−	XDR	1

HAL: Hospital Arzobispo Loayza, HNCH: Hospital Nacional Cayetano Heredia. New alleles and new sequence types described in this study are highlighted in bold. (−) negative phenotype. PS: Pan-susceptible; MR: Moderately drug-resistant; MDR: Multi drug-resistant. XDR: Extensively drug-resistant. XDR*: isolates resistant to all the antimicrobial agents tested except colistin. (1) wound/abscesses, (2) urine, (3) bronchial secretion, (4) catheter, (5) sputum.

## Discussion

This study aimed to determine the presence of the *exoU*+/*exoS*+ genotype and its association with different phenotypic and genetic characteristics, with special emphasis on MDR levels and the underlying mechanisms and efflux pump regulators in clinical isolates of *P*. *aeruginosa*. The *exoU* gene was present in 22.7% of our isolates, with a trend to be more frequent among HAL isolates, which might be explained because the observed association of *exoU* genotype with patients attending to burn ward (all from HAL). Nonetheless, the *exoU*+ *P*. *aeruginosa* showed no association with a specific source of infection. Other studies have reported that this gene was present in 28–42% of *P*. *aeruginosa* isolates causing acute infections, being especially related to pneumonia and respiratory infections^8,11,20,21^.

On the other hand, in the current study the presence of the *exoS*, *exoT* and *exoY* genes was found in 100% of the isolates. Similar results were reported for other studies in which the prevalence of these genes varied from 58–72% for *exoS*, for 89% of *exoY*, for 92–100% of *exoT*^11,22^. Interestingly, previous studies have shown the mutual exclusion of the *exoU* and *exoS* genes^5,7,10,11^. However, few studies have reported the concomitant presence of both genes in association with acute infection, being for instance detected in 40 out of 60 (67%) isolates of *P*. *aeruginosa* from bacteremia, belonging to 42 different pulse-field gel electrophoresis patterns^16^. The clonal relationships among the current analysed isolates were previously determined by Horna *et al*.^23^, with the 189 *P*. *aeruginosa* distributed in 72 different BOX-patterns; of these, 27 BOX-patterns were represented by a single isolate and the remaining 45 BOX-patterns including up to 14 isolates^23^. The *exoU*+/*exoS*+ genotypes detected in our study were distributed within 25 out of these 72 different BOX-patterns, therefore, as in the study of Morales-Espinosa^16^, the current results do not represent the spread of a successful local clone. In addition, the presence of 16 BOX-patterns containing both *exoU*+ and *exoU−* genotypes suggests genetic events of acquisition/loss of the *exoU* encoding genomic islands and of intraspecies diversity due to the dynamic nature of the accessory genome of this microorganism^24–26^. Analysis of MLST patterns in a subset of 25 isolates, also showed high clonal heterogeneity, even in the *exoU*+ isolates. In addition, 2 of these ST (ST235 and ST357) had for both *exoU*+ and *exoU−* isolates. Although unusual, the presence of the *exoU−*/*exoS*+ genotype in isolates belonging to ST235 has also been previously described^27^. This finding agrees with the proposed events of acquisition/loss of the *exoU* gene. Furthermore, the present results support the proposed *P*. *aeruginosa* non-clonal epidemic population structure^28^, both highlighting the presence of several high-risk clones (such as ST235 and ST357) with a worldwide distribution^12,25,29^, and also showing the presence of a number of undescribed *P*. *aeruginosa* ST patterns in under studied geographical areas.

It has been proposed that *P*. *aeruginosa* possessing swarming motility are more prone to presenting T3SS^30^, and some authors have related the presence of swarming and swimming as well as that of *exoU* to higher virulence^31^. Nonetheless, in our study, no specific association was observed between the presence of the *exoU*+ genotype and motility. Regarding biofilm formation, Azimi *et al*. showed that only 2.5% of the isolates presenting the *exoU* and *exoS* genes were biofilm producers and that all non-biofilm producer isolates presented the *exoU* and/or *exoS* genes^32^. In agreement with this finding, although only 1/43 *exoU*+/*exoS*+ genotype isolates were unable to form biofilm, the present results did not show an association between the presence of *exoU* and SBP.

Some studies have reported that the *exoU*+/*exoS*− genotype was found to be significantly associated with MDR compared to the *exoU−*/*exoS*+ genotype^1,2,4,33^. This relationship was not observed in our study, since the *exoS* gene was found in all the isolates. However, the *exoU*+ isolates were significantly associated with MDR and XDR when compared to *exoU−* isolates. This association between the *exoU* genotype and the MDR/XDR phenotypes may be due to the presence of transferable antibiotic-resistant determinants such as integrons carrying mobile gene cassettes within the accessory genome of *exoU*+ *P*. *aeruginosa*^25,34^.

In agreement with other studies, here, the presence of the *exoU* genotype was associated with increased levels of FQ resistance as well as with *P*. *aeruginosa* isolates displaying high MIC levels to this antimicrobial class^1,2,4,22^. Similar to Agnello *et al*.^35^, the present results agree that the development of high level of resistance to FQ has a lower fitness cost on *exoU*+ compared to *exoU− P*. *aeruginosa* isolates. Of note, the emergence of the *exoU*+ ST235, established around 1984^29^, coincides with the beginning of the use of FQ^36^, suggesting that the worldwide dissemination of this (and other) *exoU*+ ST has been favoured by this lack of deleterious effect on fitness of selected QRDR mutations^29^.

In agreement with other studies, the most frequent mutations were found in the amino acid codon 83 (T83I) and/or 87 (D87N) of *gyrA* and 87 (S87L) of *parC*^1^. In addition, a higher proportion of multiple TSM in *gyrA* and *parC* was found in *exoU*+ than in *exoU−* isolates. However, these were not significantly different, as also previously reported by Takata *et al*.^4^. Three resistant isolates had a single mutation in *parC* showing that a previous *gyrA* mutation is not a strict requisite for the acquisition of mutations at other target genes leading to resistance^1^. Finally, one isolate showed an uncommonly reported substitution in GyrA (E153K) having moderate resistance to FQ (MIC LVX = 32 mg/L, OFX = 64 µg/mL and CIP = 8 mg/L). This mutation has been previously identified in a *P*. *aeruginosa* isolate having a MIC of CIP of 8 mg/L and a concomitant amino acid change S87L in *parC*, and in two FQ-resistant unrelated *Escherichia coli* isolates, but neither data on MIC levels nor information of concomitant TSM was provided^37,38^.

*P*. *aeruginosa* has several RND-type efflux pumps, being MexAB-OprM, MexCD-OprJ, MexEF-OprN and MexXY-OprM efflux pumps well investigated^39^. In our study the *exoU*+ isolates showed higher proportions of irrelevant modifications in the regulators of MexAB-OprM and MexEF-OprN, and therefore these efflux pumps could presumably show normal basal expression levels. In agreement, in a previous study analysing *mexA* expression in a subset of isolates included in this study, those isolates having irrelevant modifications in MexAB-OprM regulators showed *mexA* expression levels equivalent to PAO1 and significant lowers (*p* = 0.02) than those reported in isolates having relevant modifications in these regulator genes^17^. Furthermore, this finding agrees with other studies showing that the isolates overexpressing *mexB* were less likely to be found among *exoU*+ isolates, and the overexpression of *mexF* and *mexD* was not correlated with the *exoU*+ genotype^4^. Although, in our study, almost all isolates showed the *mexZ* gene with irrelevant modifications (and as above may be considered as fully functional), it has been reported that the isolates overexpressing *mexY* were significantly associated with the *exoU*+ genotype^4^.

The 5 isolates possessing resistance to any of the FQ tested in the absence of a TSM were *exoU−*, further showing relevant alterations in at least one efflux pump regulator gene. Furthermore, data on 1094 isolate MexA and MexE expression levels were recorded in a previous study, showing increased *mexA* gene expression^17^. This finding suggests that in *exoU−* isolates FQ resistance will be more prone to be developed by mechanisms different to TSM supporting the proposed higher fitness cost of TSM in *P*. *aeruginosa* isolates presenting the *exoU−* genotype^35^.

Previous studies have reported that *oprD* mutations alone is the source of non-susceptibility to imipenem, and the mechanisms leading to meropenem resistance are thought to be multifactorial^40^. Although in a previous study an association between *oprD* defective mutations and the presence of *exoU* was observed^4^, this scenario was not found in the current study, also in agreement with the lack of association between *exoU* and imipenem non-susceptibility.

Regarding the presence of *β-*lactamases, non-conclusive associations were found, despite a higher proportion of *bla*_GES_ being found in *exoU*+ isolates and *bla*_IMP_ in the *exoU−* isolates. Likewise, Takata *et al*. did not find differences in the prevalence of *bla*_IMP_ between *exoU*+ and *exoU−* isolates^4^. These data together suggest that the prevalence of specific transferable genes may be more related to the specific prevalence of the gene in the area analysed than with to specific exoenzyme genetic background. Furthermore, GES-type β-lactamases and IMP-metallo-carbapenemases have been widely reported in South America, including Peru^23,41–43^.

Overall, the association between the *exoU*+ genotype and MDR/XDR was shown, despite the presence of β-lactamase, mutations in *gyrA* and *parC*, relevant modification in efflux pumps and OprD not being significantly associated with *exoU*+ isolates. Thus, the MDR/XDR phenotypic basis of the *exoU*+ genotype remains to be elucidated. One limitation of this study was that not all the mechanisms of resistance were determined, and therefore, other mechanisms might be correlated to MDR and *exoU*+ genotype.

Overall, these data suggest that *exoU*+ genotype might be genetically favoured in environments with high antibiotic pressure, such as ICUs^10^. In fact, it has been observed its adaptation to FQ-rich environment^1^. As has been commented above, the *exoU*+ isolates were more prone to be associated with ICU and burn wards, and subsequently with the most fragile patients of hospital environment. However, no data on background and final outcome of the patients was recorded, therefore lacking data about patient risk factors facilitating *exoU*+ *P*. *aeruginosa* infections, and information about patient mortality. Analysed together, these data agree with previous studies showing that *exoU*+ isolates were significantly found in man-made environmental sites while the *exoS*+ isolates were found in natural environmental sites^13,44^.

Regarding the ST detected, ST179, ST235, ST308, ST348, ST357 and ST699 are reported in the *Pseudomonas aeruginosa* MLST Database as having been previously described on different continents, being therefore widely disseminated^45^. ST235 and ST357 are among the most widespread high-risk clones. The results of the subset of isolates analysed agree with this distribution, being the two most frequently detected STs. Furthermore, the present data suggest a different hospital distribution of both STs. ST235 has been associated with a poor clinical outcome in part due to its high level of antibiotic resistance and the presence of the *exoU*+ gene^12,29^. Regarding antibiotic resistance, ST235 and ST357 are usually resistant to FQ, aminoglycosides and β-lactams^12,25,46,47^. Likewise, all the isolates belonging to these STs were at least MR, with all ST357 isolates being XDR and displaying resistance to all antibacterial agents tested except colistin. Regarding antibiotic resistance mechanisms, 3 out of 6 isolates belonging to ST235 (isolates 1077, 1078 and 1079) displayed the presence of *bla*_GES_ and an additional isolate (isolate 1080) possessed *bla*_IMP_ (Table 6). Similarly, the presence of *bla*_GES_ and *bla*_IMP_ was detected in 2 (isolates 1093 and 1105) and 1 (isolate 1089) respectively, of the isolates belonging to the ST357. In addition, all ST235 and ST357 isolates identified showed the presence of QRDR mutations.

The remaining *exoU*+ isolates belonged to ST308, ST348, ST759 and ST2726, as well as to the newly identified ST3300 and ST3303. Among these, the presence of *exoU* has been largely described on the high-risk clone ST308, which usually presents an MDR/XDR phenotype, related to a variety of molecular mechanisms including carbapenemases such as NDM^48,49^. On the other hand, ST641 has been previously reported in Korea in association with antimicrobial resistance and the *exoU−/exoS*+ genotype^50^. Data of the remaining STs are scarce, with ST179 being the most well characterised. In the present study, ST179 was found in an MDR isolate from a bronchial secretion. Accordingly, this clone has been previously associated with MDR *P*. *aeruginosa*, causing chronic respiratory infections in Spanish hospitals^51,52^.

In conclusion, an unusual high number of unrelated clinical isolates of *P*. *aeruginosa* showing the *exoU* and *exoS* genes concomitantly were found, suggesting the presence of specific pressures which facilitate the stable presence of both genes and highlight their concomitant dissemination in the area studied. The *exoU*+/*exoS*+ were associated with MDR and XDR. Furthermore, these isolates showed an enhanced ability to acquire higher levels of FQ resistance, which might be related to lower fitness cost. Rapid diagnostic determination of virulence genotypes and antibiotic resistant profiles as well as continuous surveillance are needed to monitor these high-risk *P*. *aeruginosa* isolates.

## Material and Methods

### Study area

The Hospital Nacional Cayetano Heredia (HCNH) is a level III-1 hospital with 452 beds (including a total of 24 in the ICUs, of these 6 beds belonging to a Neonatal ICU), which receives patients from around all the country^53,54^. The direct reference area is composed by the districts in the north of the city of Lima, the largest urban area of the capital, in which the population is heterogeneous: urban, rural and marginal urban. In 2011 the HCNH receives 147,642 outpatients, with 17,558 hospital admissions^53^. The Hospital Arzobispo Loayza (HAL) is also considered as a level III-1 hospital accounting for 806 beds (Of these 26 presents in ICUs)^55^. In addition, HAL acts as reference center for burn patients^56^. Its referral area comprises districts in the center of Lima, attending also population of other Lima districts and Peruvian areas, with a heterogeneous population: urban and urban marginal. In 2011 the HAL receives 218,123 outpatient visits (10.3% from outside of Lima), with 29,158 hospital admissions^57^. In 2014 the population of Lima was reportedly 9,752,000 inhabitants, 2,475,432 and 1,796,112 living in the north and center districts respectively^58^.

### Bacterial isolates

One hundred eighty-nine non-duplicated *P*. *aeruginosa* clinical isolates were recovered from December 2012 to June 2013 in two Peruvian hospitals in the course of a previous study^23^. Of these, 77 isolates were from the HAL and 112 from the HNCH.

Different characteristics were analysed previously, including clonal relationships, biofilm production, bacterial motility and antimicrobial resistance^23^. The clonal relationships were determined using DNA fingerprinting of all isolates which were generated by BOX-PCR analysis (Supplementary Figure)^23^. Finally, antimicrobial susceptibility to cephalosporins [ceftazidime, cefepime], monobactams [aztreonam], β-lactams+ inhibitors [piperacillin-tazobactam], carbapenems [imipenem, meropenem], aminoglycosides [gentamicin, tobramycin, amikacin], FQs [ciprofloxacin, levofloxacin and ofloxacin] and polymyxins [colistin] has been previously reported according to the CLSI guidelines^23,59^. Antibiotics were grouped in the above indicated categories according to Magiorakos *et al*.^60^ PS was defined as susceptibility to all the antimicrobial agents tested. MR was defined as non-susceptibility to at least 1 antibacterial agent of 1 or 2 antimicrobial categories. MDR was defined as non-susceptibility to at least 1 antimicrobial agent of three or more antimicrobial categories. XDR was defined as non-susceptibility to at least 1 antimicrobial agents in all but 2 or fewer antimicrobial categories.

### Detection of T3SS genes by PCR

The presence of the *exoS*, *exoT*, *exoU* and *exoY* genes was determined by PCR with the primers and conditions shown in Table 6. To confirm the reliability of the results, *exoS* (from *exoU−*/*exoS*+ isolates) as well as *exoY* and *exoT* amplified products were randomly selected, recovered and sequenced. Regarding *exoU*+/*exoS*+ genotype, a representative isolate from each unrelated BOX-pattern was selected and both genes were sequenced. The *exoS*, *exoT*, and *exoY* were compared with that of the *P*. *aeruginosa* PAO1 (GenBank accession no.AE004091). Meanwhile, the *exoU* gene was compared with that of the *P*. *aeruginosa* PA103 (GenBank accession no. AAC16023). In order to prevent the misidentification of the sought T3SS genes related to the high degree of sequence identity among these genes, all the primers were *in silico* tested previously against all T3SS effectors and with the full genome of the PAO1 strain.

### Analysis of the quinolone resistance determining region of* gyrA*/*parC*

The amplification of the quinolone resistance determining region (QRDR) of *gyrA* and *parC* was performed by PCR (Table 6). All the PCR products were sequenced and the QRDR of the *gyrA* and *parC* genes were compared with those of the *P*. *aeruginosa* PAO1 reference strain.

### Analysis of *oprD* and efflux regulator genes

The amplification of the *oprD* and the efflux regulator-encoding genes *mexR*, *nalC*, *nalD*, *mexT* and *mexS* were reported in a previous study^17^. The amplification of the *nfxB* and *mexZ* genes was performed with the primers designed by Solé *et al*.^61^, with slight modifications of the annealing conditions (Table 6). All the PCR products were recovered and sequenced as above. The *nfxB* and *mexZ* genes were compared with those of *P*. *aeruginosa* PAO1. Overall, amino acid substitutions, insertions and deletions were considered as an “Irrelevant modification” and frameshifts, premature STOPs, and no amplification of PCR genes was considered as a “Relevant modification” following the criteria previously described by Horna *et al*.^17^.

### β-lactamases gene detection

The presence of metallo-β-lactamases and serine-carbapenemases was determined in all the isolates by means of EDTA and boronic acid combined disc tests, respectively^62–64^. In those isolates in which the use of EDTA or boronic acid showed an increase in the disc diameter halo ≥ 5 mm, the presence of metallo-β-lactamases (*bla*_IMP_, *bla*_GIM_, *bla*_SIM_, *bla*_SPM_ and *bla*_VIM_) and serine-carbapenemases (*bla*_IMI_, *bla*_GES_ and *bla*_KPC_) was determined by PCR. Amplified products were randomly selected, recovered and sequenced as above. Table 6 shows the annealing temperature, which was slightly modified in some cases.

### Multi-locus sequence typing

A subset of 25 isolates, from the 50 for which data of antimicrobial resistance mechanisms were available, were typed using MLST. This assay was performed according to that described in the MLST database website (https://pubmlst.org/paeruginosa/) with slight modifications (Table 7). Thus, 13 *exoU*+ isolates and 12 randomly selected *exoU−* isolates were included in this analysis. All PCR products were purified, sequenced and thereafter compared with the allele sequences stored in the MLST database in order to establish the specific alleles and STs. All the isolates analysed, as well as newly detected alleles/ST profiles, were submitted to https://pubmlst.org/paeruginosa/ and are reported accordingly throughout the text.

**Table 7:** Primers used in the study

Amplified product	Primers	Sequence (5′ → 3′)	Amplicon size (bp)	Annealing Temperature	Ref.
Type III secretion system					
*exoU*	exoU - F	ATG CAT ATC CAA TCG TTG	2000	58 °C	^65^
	exoU - R	TCA TGT GAA CTC CTT ATT			
*exoS*	exoS - F	GCG AGG TCA GCA GAG TAT CG	118	58 °C	^65^
	exoS - R	TTC GGC GTC ACT GTG GAT			
*exoT*	exoT - F	AAT CGC CGT CCA ACT GCA TGC G	152	58 °C	^65^
	exoT - R	TGT TCG CCG AGG TAC TGC TC			
*exoY*	exoY - F	CGG ATT CTA TGG CAG GGA GG	289	58 °C	^65^
	exoY - R	GCC CTT GAT GCA CTC GAC CA			
Quinolone resistance determining region of *gyrA/parC* and efflux pumps regulators					
*gyrA*	gyrA - F	TTA TGC CAT GAG CGA GCT GGG CAA CGA CT	364	57 °C	^61^
	gyrA - R	AAC CGT TGA CCA GCA GGT TGG GAA TCT T			
*parC*	parC - F	ATG AGC GAA CTG GGG CTG GAT	208	57 °C	^61^
	parC - R	ATG GCG GCG AAG GAC TTG GGA			
*mexZ*	mexZ - F	CCA GCA GGA ATA GGG CGA CCA GGG C	1059	64 °C	^61^
	mexZ - R	CAG CGT GGA GAT CGA AGG CAG CCG G			
*nfxB*	nfxB - F	CGC CCC GAT CCT TCC TAT T	924	64 °C	^61^
	nfxB - R	ACG AGC GTC ACG GTC CTT T			
Metallo-β-lactamases					
*imp*	IMP - F	GGA ATA GAG TGG CTT AAY TCT C	188	55 °C	^66^
	IMP - R	CCA AAC YAC TAS GTT ATC T			
*vim*	VIM - F	GAT GGT GTT TGG TCG CAT A	390	55 °C	^66^
	VIM - R	CGA ATG CGC AGC ACC AG			
*gim*	GIM - F	TCG ACA CAC CTT GGT CTG AA	477	55 °C	^66^
	GIM - R	AAC TTC CAA CTT TGC CAT GC			
*spm*	SPM - F	AAA ATC TGG GTA CGC AAA CG	271	55 °C	^66^
	SPM - R	ACA TTA TCC GCT GGA ACA GG			
*sim*	SIM - F	TAC AAG GGA TTC GGC ATC G	570	55 °C	^66^
	SIM - R	TAA TGG CCT GTT CCC ATG TG			
Serine-carbapenemases					
*kpc*	KPC - F	GTA TCG CCG TCT AGT TCT GC	636	55 °C	^67^
	KPC - R	GGT CGT GTT TCC CTT TAG CC			
*imi*	IMI - F	ATA GCC ATC CTT GTT TAG CTC	818	55 °C	^68^
	IMI - R	TCT GCG ATT ACT TTA TCC TC			
*ges*	GES - F	GTT TTG CAA TGT GCT CAA CG	371	55 °C	^68^
	GES - R	TGC CAT AGC AAT AGG CGT AG			
Multi-locus sequence typing					
*acsA*	acsa - F	ACC TGG TGT ACG CCT CGC TGA C	524	60 °C	^45^
	acsA - R	AGG TTG CCG AGG TTG TCC AC			
*aroE*	aroE - F	TGG GGC TAT GAC TGG AAA CC	1053	60 °C	^45^
	aroE - R	TAA CCC GGT TTT GTG ATT CCT ACA			
*guaA*	guaA - F	CGG CCT CGA CGT GTG GAT GA	673	60 °C	^45^
	guaA - R	GAC GTT GTG GTG CGA CTT GA			
*mutL*	mutL - F	AGA AGA CCG AGT TCG ACC AT	705	60 °C	^45^
	mutL - R	GGG TAT AGG CGG AAT AGC C			This Study
*nuoD*	nuoD - F	GCT TCA AGC CGG AAG ACT GG	438	60 °C	This Study
	nuoD - R	TGG CGG TCG GTG AAG GTG AA			^45^
*ppsA*	ppsA - F	GGT CGC TCG GTC AAG GTA GTG G	556	60 °C	^45^
	ppsA - R	GTA TCG CCT TCG GCA CAG GA			
*trpE*	trpE - F	TTC AAC TTC GGC GAC TTC CA	603	60 °C	^45^
	trpE - R	CCC GGC GCT TGT TGA TGG TT			

bp: basepair; Ref: Reference; F: Forward; R: Reverse.

### Statistical analysis

The χ^2^ test was used for statistical analysis. P values ≤ 0.05 were considered significant. The R study version 3.4.0. was used to perform the statistical analysis. Resistant and intermediate isolates were classified together as “non-susceptible” for statistical analyses.

### Compliance with ethical standards

The study was approved by the Ethical Committee of the Universidad Peruana Cayetano Heredia (Lima, Peru) and by the Ethical Committee of Hospital Clinic (Barcelona, Spain), and all experiments were performed in accordance with relevant guidelines. All samples were obtained within routine clinical practice; no personal data was requested or available to researchers.

## Supplementary information

Supplementary Figure

## Data Availability

The datasets generated during the current study are available from the corresponding author on reasonable request.
