# Supplementary material for: High frequency of the exoU+/exoS+ genotype associated with multidrug-resistant “high-risk clones” of Pseudomonas aeruginosa clinical isolates from Peruvian hospitals
Source: Sci Rep. 2019 Jul 26;9:10874. doi: 10.1038/s41598-019-47303-4 (PMC6659710; doi:10.1038/s41598-019-47303-4)
Supplement: Supplementary file 1 — Supplementary Figure [file 41598_2019_47303_MOESM1_ESM.pdf]

Supplementary Figure

High frequency of the *exoU+/exoS+* genotype associated with multidrug-resistant "high-risk clones" of *Pseudomonas aeruginosa* clinical isolates from Peruvian hospitals

Gertrudis Horna[a,b,c], Catherine Amaro[d], Aida Palacios[d], Humberto Guerra[c], Joaquim Ruiz[a,e]

[a] Barcelona Institute for Global Health, ISGlobal, Hospital Clinic - Universitat de Barcelona, Barcelona, Spain. [b] Universidad Catolica los Angeles de Chimbote, Instituto de Investigacion, Chimbote, Peru. [c] Universidad Peruana Cayetano Heredia, Instituto de Medicina Tropical Alexander von Humboldt, Lima, Peru. [d] Hospital Nacional Cayetano Heredia, Lima, Peru. [e] Universidad Continental, Lima, Peru.

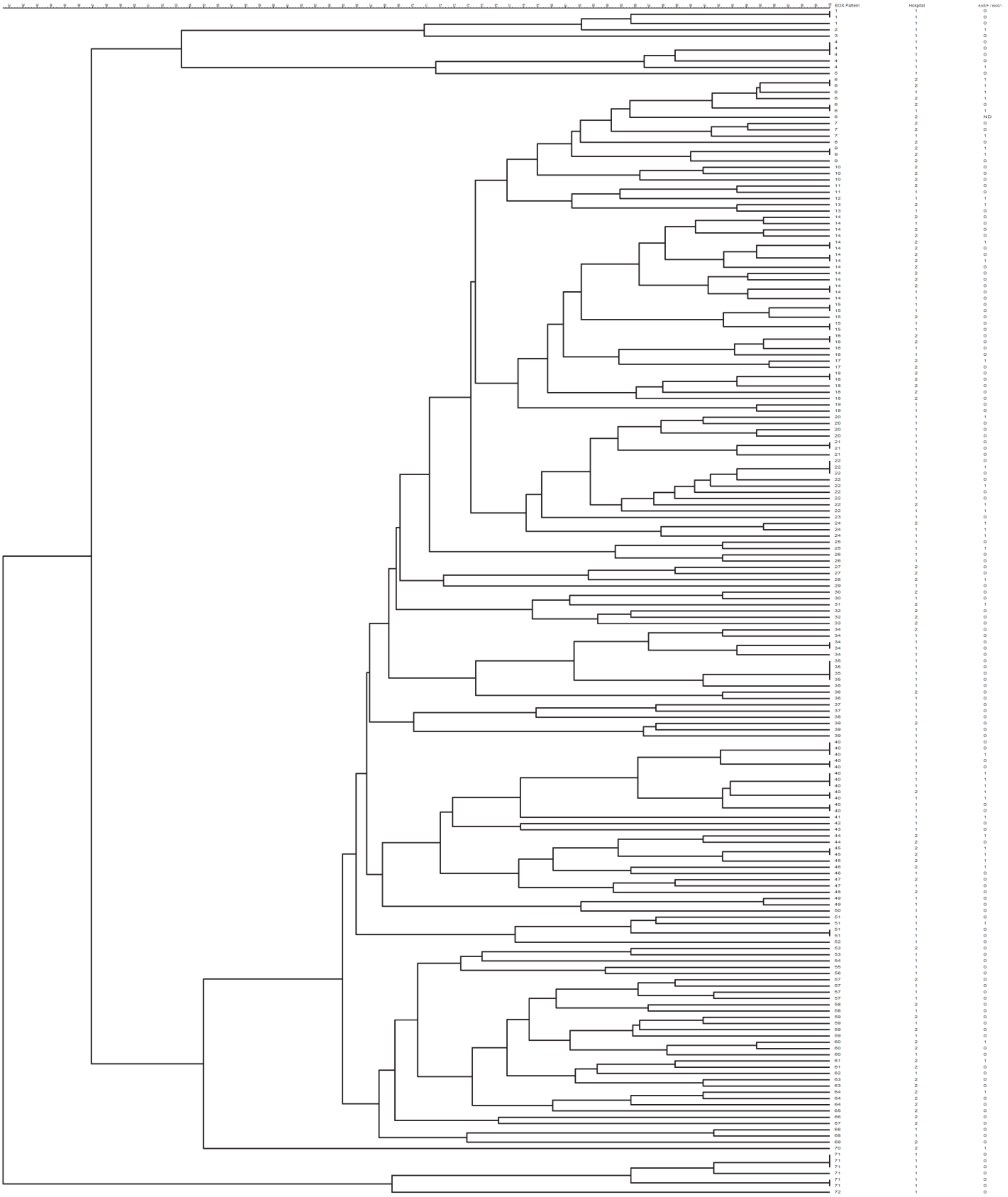
BOX Pattern
Hospital

Dendogram of the 189 clinical isolates of *P. aeruginosa* based on the BOX patterns

One isolate of the BOX-pattern 6 was not recovered from frozen stock and not included in the present study.

The isolates with ≥ 85%of similarity were considered to be related.

Hospital. 1: Hospital Nacional Cayetano Heredia; 2: Hospital Arzobispo Loayza

exoU+ = 1; *exoU* - = 0
